# Implementation of smoking cessation guidelines in the emergency department: a qualitative study of staff perceptions

**DOI:** 10.1186/1940-0640-9-1

**Published:** 2014-01-24

**Authors:** David A Katz, Monica W Paez, Heather S Reisinger, Meghan T Gillette, Mark W Vander Weg, Marita G Titler, Andrew S Nugent, Laurence J Baker, John E Holman, Sarah S Ono

**Affiliations:** 1 Department of Medicine, University of Iowa, Iowa City, IA, USA; 2 Department of Emergency Medicine, University of Iowa, Iowa City, IA, USA; 3 Department of Epidemiology, University of Iowa, College of Public Health, Iowa City, IA, USA; 4 Department of Psychology, University of Iowa, Iowa City, IA, USA; 5 Department of Anthropology, Iowa State University, Ames, IA, USA; 6 Comprehensive Access & Delivery Research and Evaluation (CADRE) Center, Iowa City VA Hospital, Suite 42-151, 601 Highway 6 West, 52246-2208, Iowa City, IA, USA; 7 University of Michigan School of Nursing, Ann Arbor, MI, USA; 8 Department of Emergency Medicine, Iowa Methodist Medical Center, Des Moines, IA, USA

**Keywords:** Emergency medical services, Smoking cessation, Attitude of health personnel, Qualitative research, Content analysis

## Abstract

**Background:**

The US Public Health Service smoking cessation practice guideline specifically recommends that physicians and nurses strongly advise their patients who use tobacco to quit, but the best approach for attaining this goal in the emergency department (ED) remains unknown. The aim of this study was to characterize emergency physicians’ (EPs) and nurses’ (ENs) perceptions of cessation counseling and to identify barriers and facilitators to implementation of the 5 A’s framework (Ask-Advise-Assess-Assist-Arrange) in the ED.

**Methods:**

We conducted semi-structured, face-to-face interviews of 11 EPs and 19 ENs following a pre-post implementation trial of smoking cessation guidelines in two study EDs. We used purposeful sampling to target EPs and ENs with different attitudes toward cessation counseling, based on their responses to a written survey (Decisional Balance Questionnaire). Conventional content analysis was used to inductively characterize the issues raised by study participants and to construct a coding structure, which was then applied to study transcripts.

**Results:**

The main findings of this study converged upon three overarching domains: 1) reactions to the intervention; 2) perceptions of patients’ receptivity to cessation counseling; and 3) perspectives on ED cessation counseling and preventive care. ED staff expressed ambivalence toward the implementation of smoking cessation guidelines. Both ENs and EPs agreed that the delivery of smoking cessation counseling is important, but that it is not always practical in the ED on account of time constraints, the competing demands of acute care, and resistance from patients. Participants also called attention to the need for improved role clarity and teamwork when implementing the 5 A’s in the ED.

**Conclusions:**

There are numerous challenges to the implementation of smoking cessation guidelines in the ED. ENs are generally willing to take the lead in offering brief cessation counseling, but their efforts need to be reinforced by EPs. ED systems need to address workflow, teamwork, and practice policies that facilitate prescription of smoking cessation medication, referral for cessation counseling, and follow-up in primary care. The results of this qualitative evaluation can be used to guide the design of future ED intervention studies.

**Trial registration:**

ClinicalTrials.gov registration number NCT00756704

## Background

Given that approximately 25 million smokers present to the emergency department (ED) annually [1], greater involvement of ED staff in cessation counseling has the potential to augment cessation rates at the population level. The US Public Health Service (USPHS) guideline specifically recommends that physicians and nurses should strongly advise their patients who use tobacco to quit, and calls for systems and practice policies to facilitate the delivery of smoking cessation counseling and pharmacotherapy [2]. Based largely on evidence from primary care settings, a public health task force convened by the American College of Emergency Physicians strongly recommends implementation of smoking cessation counseling in the ED setting [3].

Many ED patients desire preventive services for smoking cessation [4], and report interest in receiving cessation counseling during or after the ED visit [5,6]. Despite this interest, the provision of brief cessation counseling in the ED is suboptimal [7-9]. Emergency physicians (EPs) are likely to gather information about smoking, but do not consistently advise patients to quit [7,10]. EPs cite lack of time and the perception that counseling is relatively ineffective as barriers to routine cessation counseling [11]. Although the evidence demonstrates the need for better delivery of smoking cessation counseling, few published studies have investigated the feasibility and effectiveness of smoking cessation interventions using ED personnel to deliver the counseling intervention.

Interventions that facilitate the involvement of different members of the treatment team in cessation counseling are more likely to increase quit attempts by smokers [12]. Moreover, the feasibility of training ED nursing staff to deliver brief cessation counseling has been previously demonstrated [13,14]. Sustainable improvements in care, however, depend on a deeper understanding of both EPs’ and emergency nurses’ (ENs) perspectives toward smoking cessation and the treatment context (i.e., the work environment in which the practice intervention takes place) [15,16]. Thus, the main objective of this qualitative study is to characterize EPs’ and ENs’ perceptions of smoking cessation counseling, to identify barriers and facilitators to implementation of a multifaceted smoking cessation intervention, and elucidate quantitative outcomes. As the study intervention led to significant increases in performance of some (but not all) of the 5A’s, another goal of this study was to identify reactions of ED staff to the study intervention and to elicit suggestions to further improve the process of cessation counseling in this setting.

## Methods

### Study design

We used a sequential explanatory mixed methods design in which qualitative results are used to assist in explaining the findings of a primarily quantitative study [17]. Specifically, we conducted semi-structured, one-on-one interviews of EPs and ENs following the intervention period of a pre-post implementation trial of smoking cessation guidelines at each study ED. The primary aim of this trial was to determine the effectiveness of an ED implementation intervention on the use of evidence-based smoking cessation counseling strategies and quit rates in adult smokers who presented to the ED of two hospitals in Iowa. Based on the Chronic Care Model, the intervention included face-to-face training on the 5 A’s (Ask-Advise-Assess-Assist-Arrange) [18] for brief smoking cessation counseling and an online tutorial for ED nurses, use of a guideline algorithm, fax referral of motivated smokers to the state tobacco quitline for proactive telephone counseling, and group feedback to ED staff. The intervention phase lasted 3-5 months at each study site. Details of the implementation trial and quantitative findings are provided elsewhere [14,19]. This project was approved by the Institutional Review Board at each study hospital, and written consent was obtained from all subjects.

### Study setting and population

We included one University hospital that has a residency training program in Emergency Medicine (Hospital 1) and one large community teaching hospital that contracts with a group of private practice physicians to provide emergency medical services (Hospital 2) (Table 1). Both hospitals have a large annual volume of ED patients and a substantial proportion of uninsured patients. Hospital 1 used an electronic medical record (EMR), whereas Hospital 2 relied upon paper charting at the time of this study.

**Table 1:** Description of study sites

**Variable**	**Hospital 1**	**Hospital 2**
Annual ED volume	39,573	29,418
Type of medical record	Electronic	Paper-based
***Patient characteristics***		
Age (mean)	41.4	49.3
Sex (% female)	53	57
Race/ethnicity, %		
White (non-Hispanic)	88.8	89.7
Black	6.7	7.4
Hispanic	2.9	2.0
Other	1.6	0.9
Uninsured, %	10	7
Mode of transport (% private vehicle or walk-in)	78	78
ED disposition (% admitted)	25	30

All ENs and EPs who enrolled in this trial were also invited to participate in a one-on-one post-intervention interview. Of the 73 ENs and 49 EPs at both sites who participated in pre- and post-intervention assessments of the main trial, 56 (77%) and 22 (45%) consented to an in-depth interview, respectively. “Float” nurses were excluded because they were much less likely to have had exposure to the study intervention; nursing assistants were excluded because they are typically not involved in providing patient education.

**Figure 1:**
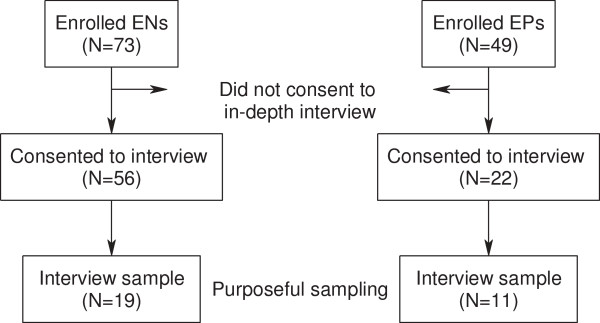
Recruitment of interview sample.

To ensure variability in pre-intervention attitudes toward cessation counseling, we used purposeful sampling to target EPs and ENs. Just prior to intervention training, both groups were administered a written survey to assess their self-efficacy and attitudes toward smoking cessation counseling (“pros” and “cons”) using the Decisional Balance Questionnaire (DBQ) [20]. Consistent with the Transtheoretical Model of Change, decisional balance has been shown to be associated with physicians’ stage of readiness to provide smoking cessation counseling [21] and with delivery of smoking cessation assistance by primary care providers [22]. Specifically, we dichotomized EPs and ENs based on their DBQ “pros” and “cons” subscale scores (dichotomized at the median). To ensure that ED staff were sampled across the spectrum of attitudes and beliefs toward cessation counseling, we then interviewed a convenience sample of EPs and ENs from four possible DBQ subgroups; 1) high pros/high cons, 2) high pros/low cons, 3) low pros/high cons and 4) low pros/low cons. In total, 19 ENs and 11 EPs completed the interview (Figure 1).

### Study protocol

A trained MA or PhD-level interviewer contacted EPs and ENs to arrange a suitable time for the study interview. Both interviewers had a strong background in the use of qualitative methods and were trained to use specific interview procedures for this study; both were unfamiliar with any members of the ED staff. An interviewers’ guide was designed to elicit perceptions about smoking cessation in the ED (see Supplemental Appendix). Each interviewer conducted practice interviews on one nurse and/or physician who were not included in the purposeful sample. Individual face-to-face interviews were conducted on-site in the ED and lasted an average of 18 minutes (range 6-31 minutes). The interviewer asked each EP and EN to describe his/her usual (pre-intervention) smoking cessation practices, to discuss his/her perceptions of the study intervention, and to discuss his/her perceptions of the role of ED staff in addressing smoking cessation. EPs and ENs were also asked if they expected to use the 5 A’s approach following study completion. We conducted interviews until saturation was attained (i.e., until no new themes were identified) [23].

### Data analysis

Interviews were audio recorded, transcribed, audited for completeness and accuracy, and imported into NVivo, a qualitative data management and analysis software program [24]. We used conventional content analysis to inductively characterize the issues raised by study participants and to construct a provisional coding structure that was then tested using a subset of transcripts [25]. To develop the codebook, a set of transcripts was open coded by three independent coders; emergent themes were discussed by the group. The codebook design grouped together similar themes or topics under overarching domains. Relevant text identified in interview data was then coded accordingly; data could be coded in more than one category. Inter-rater agreement was checked at two intervals and any discrepancies were discussed and resolved using coding consensus (Table 2) [26]. In all cases, consensus was reached and the discrepancies were resolved by clarifying code definitions, which were incorporated into the codebook and recorded in NVivo. The coding structure was revised iteratively as new themes emerged. We used coding frequency to identify thematic patterns in the data. Also, we analyzed the convergence and divergence of EPs’ and ENs’ perspectives on smoking cessation practices and their reactions to the intervention at each site.

**Table 2:** **Inter-rater agreement of qualitative coders**^**1**^

	**Hospital 1 coding group**^**2**^		**Hospital 2 coding group**	
**Staff role**	**Set 1**	**Set 2**	**Set 1**	**Set 2**
**EP**	97%	96%	94%	95%
**EN**	96%	97%	95%	98%

^1^Percentage agreement across two independent coders was calculated as an overall average of all codes. Each set consisted of unique transcripts that were each coded by a pair of independent coders (coding group). Agreement for Set 1 was determined after codebook development and included transcripts from the first 10% of coded interviews; inter-rater agreement for Set 2 was based on the last 10% of coded interviews.

^2^Three independent coders worked in paired combinations; one coder was included in each pair throughout the entire coding process to ensure consistency.

## Results

Table 3 shows the characteristics of the ED staff who completed the in-depth interview. ENs and EPs had worked in the ED for a median of 7 years. Of the nineteen nurse participants, seven were diploma-educated registered nurses (RNs) and seven had attained a bachelor’s of science in nursing (BSN); approximately half of the EPs (5 of 11) were board-certified in emergency medicine. Three of 19 ENs and none of the EPs were current smokers.

**Table 3:** **Descriptive characteristics of emergency nurses and physicians who completed in-depth interviews**^**1**^

**Characteristic**	**EN (n = 19)**^**2**^	**EP (n = 11)**^**3**^
Age, mean (sd)	40.1 (10.3)	39.6 (10.2)
Gender, % male	26	64
Race, % white	84	82
Diploma-educated RN or BSN, %	74	NA
Board certified in emergency medicine, %	NA	45
Total experience (years), median (IQR)	8 (2-9)	10 (1-16)
ED experience (years), median (IQR)	7 (1-7)	7 (1-14)
Smoking status, % current smoker	16	0

^1^RN = Registered nurse, BSN = bachelor’s of science in nursing, NA = not applicable, IQR = interquartile range. The physician category includes a small number of physician’s assistants at each site.

^**2**^53 and 47% of EN interviewees were employed at Hospitals 1 and 2, respectively.

^**3**^46 and 54% of EP interviewees were employed at Hospitals 1 and 2, respectively.

The main findings of this study converged upon three overarching domains: 1) reactions to the intervention; 2) perceptions of patients’ receptivity to cessation counseling; and 3) perspectives on ED cessation counseling and preventive care. Four of the most frequently coded themes occurred in the first domain (feedback on performance, concerns regarding post-ED follow-up, prescription of pharmacotherapy for smoking cessation in the ED, and ED barriers to providing cessation counseling). Three of the most frequently coded themes occurred in the third domain (role of the ED in providing preventive care, specific roles of ED staff in providing cessation counseling, and recommendations/suggestions for improvement). We explore each of the overarching domains in more detail below.

### Reactions to the Intervention

A representative sample of quotes by ENs and EPs regarding their reaction to the intervention and its impact on smoking cessation practice is presented in Table 4. ENs at both sites reported that implementation of the 5 A’s clinical reminder affected the following changes in their own practice: (1) increasing their awareness of smoking cessation; 2) making sure that every patient was asked about his or her smoking status; and (3) expanding their smoking history inquiry by asking more than “do you smoke?” In particular, both ENs and EPs found that the 5 A’s study algorithm provided a systematic approach to assessment and action:

*“[To] be honest with you, I see no other way to really do it and keep people on task without something to remind them, because there’s so many things going on all at once. Like times in the emergency room that people don’t have the reminder, they won’t remember to do it.”* [Nurse, Hospital 2]

*“I think the steps were very well thought through and if you follow the steps it was very easy to address the situation with patients. I think that often times we do those things somewhat randomly and not always in that order, but to have things listed in order makes the intervention much easier.”* [Physician, Hospital 1]

**Table 4:** Selected EN and EP responses to queries about changes in their smoking cessation practices during the study intervention

	**EN responses**	**EP responses**
**Hospital 1**	[250] “Well, yeah, because we would never ask about smoking prior to that….The PAs [physicians assistants] a lot of times would ask, the residents…would ask, but not much of the—not much from the nurses.”	[105] “I feel like [the intervention] is worth doing…I feel like a really big part of our job is to prevent people from getting sick, instead of just carrying them when they are sick.”
	[261] “Oh no, before the training? We never--I mean, we just wrote ‘em down, yep they smoke, that’s as far as we went, before….No, this has totally brought [smoking cessation] to our attention.”	[109] “It really takes a mindset change for emergency physicians to think about any preventative care. It’s sort of drilled into the residents and students that your goal is to get people out the door. To patch ‘em up and get ‘em out, instead of doing anything preventative necessarily. And so, in some ways the training needs to change.”
	[288] “I have found that I’m a lot more comfortable and I can—you know, the first few people that shut me down I was like, ok, that’s nice, I’m gonna just go on to the next question. But I kinda gotten more confident with talking to people and getting the information out, so it’s—it has changed because I think, I’m more willing, maybe, to keep going on the conversation…. I’ve got that back-up information right smack dab in my room. I don’t have to go anywhere, um, forget about it and go, ‘oh shoot, I forgot that person.’ It’s right there and it’s an immediate thing that you can do.”	[118] “I think it’s a bit of a change from the way the ERs have operated in the past, I think there was some resistance to this [intervention] from some of the nursing staff primarily. That’s what I remember. Mainly because of the time constraints involved, I mean we’re extremely busy. It’s kind of hard to sit down and talk to patients about tobacco cessation …. the less busy we are, the more we can implement some of these things.”
		[150] “[T]here are so many things that you’d like to address or that would be useful to address, but you have 6 or 8 patients waiting and so we don’t have the leisure of talking about everything that needs to be addressed. You have to identify those things that the patient is going to be most receptive to and talk about those things.”
**Hospital 2**	[505] “Prior to the study we didn’t do anything. Since the study though, we use the algorithm, and ask the additional questions on the algorithm, and if they show an interest in quitting, then we do make--, have been making referrals to Quitline and providing them with any information that they might need.”	[805] “I think [the intervention] was kind of hit and miss. I mean, it depended on the day, it depended on who was doing triage, it depended on the providers that were around, whether or not it got done.”
	[540] “I’m a big anti-smoker. So probably not a whole lot [of change], because I always did ask patients. And I was one of those that sort of lectured. I would you know, ‘really need to quit smoking’ and that kinda thing because I just hate smoking. But as far as being more aware and filling out the form, all that, yeah it has made a difference.”	[890] “I’d say most of the time people weren’t very receptive to it. Some got angry--if you’d ask them: think about quitting? or do you wanna quit?--or very defensive, but I would say, if a few people did think it was a good idea and they’d want information on it, even one or two would be, I suppose, successful.”
	[554] “[during intervention] I’d ask them how long they’ve smoked, have they ever tried to quit smoking. That was about it….I never asked them those questions before. And now I kind of, you know, you can tell when a person comes in…[it’s] a gut feeling.”	[878] “I do believe that we have opportunities that are perhaps less dramatic than other things that we do, but equally or more important. And this would be one of them.”
	[616] “[Now] when I ask, I go to the extent of asking, ‘Well, do you wanna quit?’ And, [that’s] something I had never done before. ‘Cause I didn’t care.”	[927] “I think that a lot of the providers `and] nurses weren’t really on board, it was kind of like, ‘Look, we don’t have time for this, this really is out of the scope of our practice.”

Time pressure and competing demands in the ED were frequently cited as barriers to cessation counseling by both ENs and EPs, however. Although both EPs and ENs supported the intent of the intervention, time constraints often trumped ideals. As a result, discussion of smoking cessation was often reported to be cut short. In addition, some staff were wary of discussing smoking cessation because of concern that it could open a “Pandora’s box” of medical and/or psychosocial ills. The following quotes expand on these barriers to implementing the intervention:

*“It wasn’t too bad. It was depending on what else was going on. I mean if I was really busy sometimes it was hard to even just stop to think, to continue to go on with it, you know. Especially nights where it was really busy and we were trying to get somebody in and out quick, you just kind of ask them if they smoke, and didn’t really go too much--- I mean, how much they smoke and if they’re willing to quit, that’s kind of a big gateway opener.”* [Nurse, Hospital 2]

*“I think initially the staff, whether it was the emergency providers [or] the nurses, we really tried to embrace it and tried to practice it, but after a while it was just somewhat overwhelming, especially on really busy days.”* [Physician, Hospital 2]

Constraints of the ED setting were identified as the main limitation to implementing the intervention, even for staff who stated that smoking cessation counseling was an appropriate intervention in the ED. Logistical concerns were context-specific and varied by site. Because the two sites used different methods for documentation, ENs had differing opinions on the practicality of providing smoking cessation counseling in the ED. The use of a paper charting system and standardized ED assessment forms at Hospital 2 (which could not be adapted for this study) was reported to be a barrier to intervention. For example, one EN explained why she did not expect to continue using the 5 A’s during the post-intervention period:

*“There’s a very minimal amount of room where we chart the smoking. There’s not any place for extra additional information, so then you’re squeezing it in, or there’s really no room, so then you can’t read the writing anyway.”* [Nurse, Hospital 2]

Overall, the intervention was regarded as well conceived and the 5 A’s were viewed as a useful tool for providing smoking cessation counseling and creating a plan of action; however, many ENs and EPs perceived significant barriers to integrating the intervention into their practice because of workflow demands in the ED and other logistical barriers.

### Perception of patients’ receptivity to cessation counseling

Another commonly mentioned barrier to providing cessation counseling was the perception that ED patients were not interested in (and were sometimes defensive about) getting advice to quit. In one case, the EN was reluctant to ask about smoking after getting rebuffed by a patient:

*“Well, they, they come in for, I don’t know, abdominal pain or something, and I’d ask ‘em if they smoke, and then if they’re ready to quit, and they’d say--, they’d just stop me right there and say ‘That’s not why I’m here. I’m here for this reason, and if I came in and wanted to quit smoking then I’d ask you.’”* [Nurse, Hospital 2]

ENs were more likely than EPs to talk about the patients’ role and responsibility in smoking cessation—often identifying this as a barrier to successful cessation counseling. Some ENs were skeptical about the utility of providing cessation counseling in the ED and were not interested in helping patients make the case for change:

“…*My personal thoughts are that people don’t take responsibility for their own actions. So, they can always blame it on something else, and I think that’s the mindframe that, you know, they do that—‘Oh, I overeat because it’s in my genes,’ you know, ‘I can’t exercise cause I’m too’-- you know, or whatever. ‘I drink cause of this’ and blah blah blah. And they don’t take responsibility for their stuff. And I think, you know, comin’ from that mind-frame…if a person’s not willing to take responsibility, then there’s nothin’— we can’t hold their hand.*” [Nurse, Hospital 1]

EPs found patients to be variably receptive to cessation counseling, and tended to advise patients to quit smoking selectively, based on non-verbal cues, the presence of smoking-related comorbidities, and how much time they had. Although the study team emphasized principles of motivational interviewing (such as exploring ambivalence to smoking or “rolling with resistance”) [27,28], EPs tended to revert to old habits, such as confronting patients about their smoking and using scare tactics. One physician explained his approach to cessation counseling as follows:

“…*You get kind of the eye roll, you’re like, ‘OK, well I’m not gonna push it. There’s no point in kind of damaging our relationship over this if you’re not receptive to it’…If they come in for something like chest pain or cough or hard time breathing, then I really lay into them actually, especially asthmatics or people with lung disease,…especially young people with [these conditions], that’s annoying, I really kind of get on ‘em…It’s really frustrating actually, and you think, ‘you’re killing yourself.’*” [Physician, Hospital 1]

### Perspectives on ED cessation counseling and preventive care

Both ENs and EPs acknowledged that the role of emergency departments is changing and that the ED encounter may provide “teachable moments” that facilitate smoking cessation counseling. In response to the intervention, some interviewees discussed the function of emergency departments in providing preventive care, arguing that the ED is an appropriate place for smoking cessation counseling because of the high patient volume. In addition, ENs acknowledged their role in patient education, including the use of informational pamphlets to assist patients with quitting.

*“I think our role is to put the information out there and at least get the people to start thinking. You know, because we see the people on an episodic manner for the most part, uh, it’s really hard to follow up with them. Um, but I think just providing the information and getting them to think about things might make a difference the next time they visit a private physician.”* [Nurse, Hospital 2]

One area that emerged from the data was the perceived difference in roles and responsibilities for delivering the smoking cessation intervention. EPs often expected ENs to take the lead on smoking cessation counseling, while ENs reported a similar expectation of EPs. EPs tended to believe that ENs had more patient contact and more time to execute the 5 A’s, thus making them better suited to delivery of the intervention:

“*As providers, we were kind of at the point where if the nurses weren’t filling out the [5 A’s] algorithm, we weren’t gonna do it. Because then that just added on a whole ‘nother line of questioning for it and it was just too much to do with everything else that you were trying to do. So, if I didn’t have a completed form or if they just had checked off they’re a smoker and this is how much and how long, I was like, ‘Well, great’, I’m not asking them anything else because this form’s not done so I don’t know if they want my intervention and I’ll ask-- I’ll tell ‘em to quit smoking, I’m not gonna go through all the rest of it with them*.” [Physician, Hospital 2]

Whereas EPs identified ENs as having more time with patients to administer the 5 A’s, ENs ascribed significant responsibility to EPs because of their unique role in prescribing medications. In contrast, EPs expressed concerns about prescribing in a setting where follow-up is unpredictable, as illustrated by the following quotes:

*“One of the major barriers is that a lot of emergency physicians feel uncomfortable writing prescriptions for a long term med. Especially things like Chantix or Bupropion might have significant side effects.”* [Physician, Hospital 1]

*“I think we have a role in educating people about smoking, and drinking, and drugs. That’s not good for your health regardless of your medical conditions. But I don’t—me personally as a clinician—I don’t feel like the emergency department is the place for starting someone on medication.”* [Physician, Hospital 2]

EPs also considered their role in providing cessation counseling in the larger context of healthcare. For example, EPs identified the need for improved coordination between ED providers and primary care with regard to assisting patients with smoking cessation, which seemed to underlie their concerns regarding pharmacotherapy.

*“I think we’re taking on [a role in preventative health]. I think we’re taking on more primary care medicine than emergency rooms should be doing, and I don’t know that, that is the best thing for the health care system, but that’s the way things are going.”* [Physician, Hospital 2]

ENs and EPs offered several practical and creative suggestions for improving the process of delivering smoking cessation counseling in the ED: 1) encouraging patients to read smoking cessation materials or view an educational video while waiting for the EP; 2) training staff to better time cessation counseling during the ED encounter (e.g., when the patient is not “writhing in pain”); 3) using an alert at discharge to notify EPs that the patient showed interest in making a quit attempt; 4) incorporating Quitline referral in the discharge orders (and simplifying the Quitline referral process); 5) providing smoking cessation medications to patients in the ED at the time of discharge (as suggested by others) [29]; and 6) having a designated ED health educator or social worker to provide more intensive cessation counseling and follow-up. EPs also recommended further streamlining the intervention such that the assessment could be completed in less time (in approximately 1 minute).

## Discussion

Smoking causes more deaths each year than alcohol, motor vehicle accidents, suicide, AIDS, homicide, illicit drugs, and fires combined [30]. Previous studies have demonstrated that it is feasible for ED staff to improve delivery of the 5 A’s [7] and that these improvements are accompanied by enhanced self-efficacy and role satisfaction in cessation counseling [14]. Indeed, opportunistic cessation counseling is strongly recommended by professional societies of emergency medicine [1]. The current study shows that ED staff acknowledge the potential public health benefit of brief cessation counseling in the ED, which serves a disproportionate number of smokers [31], but are ambivalent toward counseling patients themselves on account of time constraints, competing demands, and perceived resistance from patients.

What emerged from this study are two parallel and related conversations. The first is a direct response to the intervention and the particular tasks that emergency staff were asked to incorporate into their personal practice. The second is a broader discussion of the perceived or desired role of ENs and EPs in delivering preventive care in the ED setting. Although ENs and EPs spoke about barriers to delivering smoking cessation counseling with similar frequencies, ENs tended to raise more immediate concerns regarding the logistics of implementation whereas EPs tended to raise concerns about whether or not preventive medicine has a place in the ED altogether. These findings complement survey data showing that ENs had significantly less negative attitudes toward smoking cessation counseling in the ED compared to EPs [14]. In a recent national survey, over half of EPs did not feel that smoking cessation counseling was an appropriate service to offer in the ED, and 77% felt that smokers who want to quit should get help from their primary care providers [32].

In contrast, ENs and EPs in the current study agreed that the delivery of smoking cessation counseling is important, but that it is not always practical in the ED on account of time constraints and the competing demands of acute care [11,32]. Both groups readily identified the potential value of providing smoking cessation counseling to patients who present with health concerns that can be directly attributed to smoking [5]. They also pointed to several structural and administrative barriers, however, that may impede the integration of tobacco treatment into ED care. For example, professional practice concerns related to prescribing smoking cessation medication (given the uncertainty that patients will receive follow-up) presented a barrier to moving beyond assessing tobacco use in the ED. In addition, our results indicate the need for greater attention to role clarity when implementing the 5 A’s in the ED.

Many of the same issues raised by ED staff have also been identified as barriers to providing smoking cessation counseling in primary care (e.g., lack of patient interest, time, and organizational support) [33]. Systematic reviews in primary care have shown that failure to provide brief physician counseling translates into lower cessation rates [2,34]. Like their EP counterparts, general practitioners (GPs) report relying on body language (e.g., posture, eye contact) to judge motivation to quit and having a limited repertoire for dealing with unmotivated smokers [35]. Many GPs have not received training in motivational interviewing and describe using confrontational strategies with more resistant smokers who had significant smoking-related morbidity, although very few felt that frightening patients was effective in persuading them to make a quit attempt [35].

Limitations of this study deserve comment. First, the study was limited to two ED settings located in the upper Midwest. The ED staff and the patients they served were predominantly Caucasian. Second, the sampling strategy was based on pre-intervention decisional balance categories (using DBQ scores), and the interview data did not strongly differentiate between DBQ subgroups. Greater variability in the reactions of ED staff toward the intervention may have been attained by sampling subjects based on *post-intervention* DBQ scores. Third, the use of different interviewers at each site may have introduced subtle variation into the process of data collection. Fourth, the frequency of themes mentioned by participants was in part driven by the priorities for discussion in the interviewer’s guide. Finally, participants often had limited time available for interviews. This sometimes resulted in very brief interviews, depending on the day and time of the interviewer’s visit.

Integrating smoking cessation counseling into emergency department practice drew a mixed reaction from ENs and EPs at both sites. Time constraints, logistical factors, and role clarity in the delivery of smoking cessation counseling were identified as barriers that adversely affected implementation. Despite these challenges, both ENs and EPs have an important role in addressing tobacco dependence, akin to their widely accepted role in screening and treatment of alcohol misuse. ENs were generally receptive to taking the lead in providing brief cessation counseling, but several steps should be taken for successful implementation in the ED: 1) improve self-efficacy: provide staff training in motivational interviewing principles to offer brief cessation counseling that is appropriate for the patient’s readiness to quit (although this requires a substantial time commitment); 2) enhance EP-EN collaboration: emphasize the complementary role of ENs and EPs, the importance of teamwork in providing preventive care, and the fact that the 5 A’s are a logical extension of already established roles (e.g., the EN’s role as patient educator); 3) streamline the process of care: delivery of the 5 A’s needs to account for the EN’s workflow and possibly needs to be simplified (e.g., ask-advise-refer model) [36]; and 4) normalize the prescription of smoking cessation pharmacotherapy: EPs should be encouraged to prescribe medication for nicotine dependence in the same way that they prescribe new medications for hypertension, diabetes mellitus, or other chronic conditions.

Performance of the 5 A’s can be significantly enhanced when emergency medicine clinicians and nurses share the responsibilities of cessation counseling using a team-based approach [14]. The lessons learned from this qualitative evaluation can guide the development of future smoking cessation interventions that can be delivered by ED staff. Healthcare administrators and managers should implement ED systems and practice policies that simultaneously facilitate the delivery of smoking cessation treatment in the ED and enhance follow-up in and coordination with primary care.

## Abbreviations

5 A’s: Ask-Advise-Assess-Assist-Arrange (framework for brief smoking cessation counseling); ED: Emergency department; EN: Emergency nurse; EP: Emergency physician; DBQ: Decisional Balance Questionnaire.

## Competing interests

The authors have no potential competing interests to disclose. The views expressed in this article are those of the authors and do not necessarily reflect the position or policy of the Department of Veterans Affairs or the United States government.

## Authors’ contributions

Study concept and design: DK, HR. Acquisition of the data: MG, JH. Analysis and interpretation of the data: SO, MP, DK. Drafting of the manuscript: DK, SO. Critical revision of the manuscript for important intellectual content: DK, SO, HR, MVW, MT. Statistical expertise: JH. Obtained funding: DK. Administrative, technical, or material support: JH, AN, LB. Study supervision: SO, DK. All authors read and approved the final manuscript.

## Prior presentation

Society of Medical Decision Making Annual Meeting, Toronto, CA, October 21, 2010.
